# Lung ultrasound in infants with bronchiolitis

**DOI:** 10.1186/s12890-019-0925-4

**Published:** 2019-08-24

**Authors:** Danilo Buonsenso, Anna Maria Musolino, Antonio Gatto, Ilaria Lazzareschi, Antonietta Curatola, Piero Valentini

**Affiliations:** 1 Fondazione Policlinico Universitario A. Gemelli, IRCCS, Rome, Italy; 2 Universita Cattolica del Sacro Cuore, Roma, Italy; 3 Department of Pediatric Emergency, Bambino Gesu Children’s Hospital, IRCCS, Rome, Italy

**Keywords:** Lung ultrasound, Pneumonia, Bronchiolitis, Infants, Children, Precision medicine, Personalized medicine, Radiomics

## Abstract

Lung ultrasound (LUS) is nowadays a fast-growing field of study since the technique has been widely acknowledged as a cost-effective, radiation free, and ready available alternative to standard X-ray imaging. However, despite extensive acoustic characterization studies and documented medical evidences, a lot is still unknown about how ultrasounds interact with lung tissue. One of the most discussed lung artifacts are the B-lines [in all ages] and the subpleural consolidations (in young infants). Recently, LUS has been claimed to be able to detect pneumonia in infants with bronchiolitis, although this can be an overestimation due to the peculiar physiology of small peripheral airways of the pediatric lung (particularly in neonate/infants). Distinguishing consolidations from atelectasis in young infants with bronchiolitis can be challenging and those criteria well defined for adults and older children (size and bronchogram) cannot easily translated in this specific subset. Therefore, if decades of studies clearly defined the low risk of SBI in bronchiolitis, we need to be careful before stating that LUS may confirm pneumonia in such a high number of cases and, importantly, new and promising techniques such as LUS should give us new insights bringing us to improvements and not back to overuse of antibiotics. More studies are surely need on this topic.

## To the editor

We read with interest the manuscript “Lung ultrasound for the diagnosis of pneumonia in children with acute bronchiolitis” by Biagi et al. [1].

The study, methodologically well done, evaluated 87 children with bronchiolitis of which 25 (29%) received a final diagnosis of concomitant pneumonia made by both lung ultrasound (LUS) and chest X-ray (CXR), with a high concordance between the techniques. This is a hot topic since this represents a common diagnostic dilemma for the paediatrician and therefore deserves attention. Sensitivity and specificity of LUS for the diagnosis of pneumonia were 100 and 83.9% respectively, wsith an area under-the-curve of 0.92, while CXR showed a sensitivity of 96% and specificity of 87.1%. When only consolidations > 1 cm were considered consistent with pneumonia, LUS specificity increased to 98.4% and the sensitivity decreased to 80.0. Interestingly, as shown in Table 1 of Biagi’s manuscript [1], the 25 children with pneumonia were all those patients with moderate to severe bronchiolitis clinical score.

Stating that about 1/3 of patients with bronchiolitis have pneumonia and all those with moderate to severe bronchiolitis clinical score have concomitant pneumonia, represents a strong message that brings the potential risk of importantly rise the indiscriminate use of antibiotics in infants with bronchiolitis. This is a message that need clarifications, particularly in the era of the dangerous increase of antimicrobial resistance and renewed importance of antibiotic stewardship.

Important clinical studies, including 1 randomized trial, suggested that children with suspected lower respiratory tract infection who underwent radiography were more likely to receive antibiotics without any difference in outcomes [2, 3]. The American Academy of Pediatrics (AAP) [4], in fact, states that CXR should be reserved for severe cases needing intensive care unit admission or where signs of an airway complication (such as pneumothorax) are present.

Despite randomized controlled trials [5, 6] showed no benefit from routine antibacterial therapy for children with bronchiolitis, antibiotic therapy continues to be overused in young infants with bronchiolitis because of concern for fever [7], young age [8] and secondary bacterial infection [9]. Studies [10, 11] have shown that a child with a clear viral syndrome, such as bronchiolitis, has a much less than 1% risk of serious bacterial infection (SBI) concluding that routine screening for SBI among hospitalized febrile infants with bronchiolitis is not justified. Several retrospective studies [12–18] and four prospective studies of SBI in bronchiolitis and/or respiratory syncytial virus (RSV) infections also confirmed this [19–23].

Therefore, general literature is much distant from the article by Biagi et al. [1]. Nevertheless, considering the LUS use in this condition, we want to share our experience in LUS in infants with bronchiolitis to further reply.

We agree with Biagi et al. [1] with the potential role of LUS in bronchiolitis; in our experience we are having opposite results that remark the usefulness need for antibiotic in these patients and that LUS may be important in ruling-out pneumonia instead of confirming it from doubtful CXR.

We already published 3 articles on LUS in bronchiolitis [24–26] and we found no cases of pneumonia in these children, despite we evaluated 138 cases in the two published series.

Moreover, we are now part of an Italian multicentric study on this issues whose results will be available in about 2 years.

**Fig. 1:**
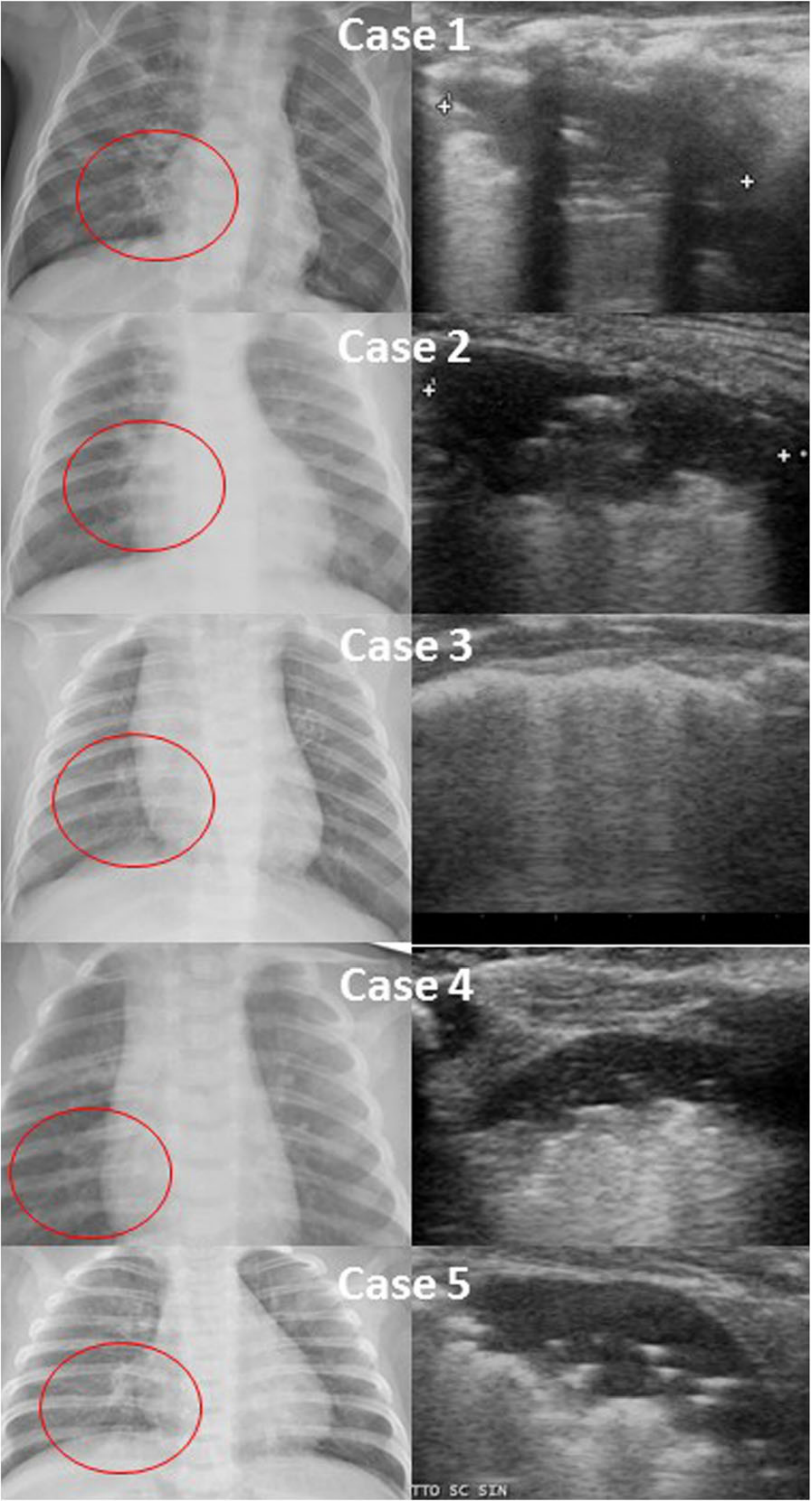
A case series of paracardiac consolidations on chest X-ray and the corresponding pattern on lung ultrasound, showing posterior, paravertebral, subpleural consolidations

We are now routinely following up on an almost daily base infants with bronchiolitis through LUS. This is part of a study approved by the Institutional Review Board of our institution, and written informed consent was obtained by both parents of our patients. To reply Biagi et al., we report the last 20 cases of RSV confirmed bronchiolitis evaluated during this ongoing epidemic season. 100% of these patients had subpleural consolidations and air bronchogram was described in 19% of cases, and almost all patients [96%] had consolidations located in the posterior paravertebral area; importantly, these consolidations sometimes disappeared the day after and in other cases persisted (although slightly reduced in size) up to 5-7 days after the first LUS independently from which treatment was performed (from high-flow nasal cannulae to antibiotics to steroids) or no treatment at all started. Most patients had a positive CXR according to the radiology on duty (Fig. 1, cases 1 to 4). LUS findings were similar despite fever and C-reactive protein levels. In one case we diagnosed a concomitant pneumonia and a microbiological confirmation of *H. influenza* pneumonia was obtained (Fig. 1, case 5). The child also had 37,070/mmc white blood cells with 20,870/mmc neutrophils and C-reactive protein 5 times higher than threshold. Interestingly, LUS appearance of this case was similar to the other non-complicated cases. This highlight that LUS semeiotic in young infants still need to be studied and clarified and that clinical findings are important for the proper interpretation of LUS findings. In our experience, whenever we face with CXR suspected for pneumonia in an infant with bronchiolitis (usually requested by paediatricians not experienced in LUS), we perform LUS to rule-out pneumonia and we are extensively reducing the use of antibiotics, as suggested by the AAP [4]. Only with extensive consolidations and abnormal laboratory findings we diagnose a concomitant pneumonia. We think these data need to be considered otherwise other paediatricians could be keep using indiscriminate empirical antibiotics in bronchiolitis.

“LUS is nowadays a fast-growing field of study” since the technique has been widely acknowledged as a cost-effective, radiation free, and ready available alternative to standard X-ray imaging [1]. However, despite extensive acoustic characterization studies [27–33] and documented medical evidences [34–44], a lot is still unknown about how ultrasounds interact with lung tissue [45]. One of the most discussed lung artifacts are the B-lines [in all ages] and the subpleural consolidations (in young infants). For example, to further study B-lines, Demi et al. [45] developed lung-phantoms by trapping a layer of microbubbles in tissue-mimicking gel. They demonstrated how the frequency of B-lines could provide a quantitative-measure of the state of the lung but, on the other hand, also highlights the heterogeneity of artefacts.

The reason for our hypothesis is that the small peripheral airways of the pediatric lung (particularly in neonate/infants) can easily change during physiological breathing and have peculiar changes during pathology. Distinguishing consolidations from atelectasis in young infants with bronchiolitis can be challenging and those criteria well defined for adults and older children (size and bronchogram) cannot easily translated in this specific subset. Therefore, if decades of studies clearly defined the low risk of SBI in bronchiolitis, we need to be careful before stating that LUS may confirm pneumonia in such a high number of cases and, importantly, new and promising techniques such as LUS should give us new insights bringing us to improvements and not back to overuse of antibiotics. More studies are surely need on this topic.

## Data Availability

The datasets used and/or analysed during the current study are available from the corresponding author on reasonable request.

## Abbreviations

AAP: American Academy of Pediatrics
CXR: Chest X-ray
LUS: Lung ultrasound
RSV: Respiratory syncytial virus
SBI: Serious bacterial infection
